# Supplementary material for: Identification of Freezing-Responsive microRNAs and Their Targets in Chinese Jujube by Small RNA and Degradome Sequencing
Source: Int J Mol Sci. 2026 Sep 4;27(17):7912. doi: 10.3390/ijms27177912 (PMC13566890; doi:10.3390/ijms27177912)
Supplement: Supplementary file 1 [file ijms-27-07912-s001.zip › Supplementary Material S2/novel_miR_158.pdf]

Provisional ID	:	novel_miR_158
Score total	:	1014
Score for star read(s)	:	3.9
Score for read counts	:	1012.1
Score for mfe	:	0.2
Score for randfold	:	-2.2
Score for cons. seed	:	
Total read count	:	1997
Mature read count	:	237
Loop read count	:	1757
Star read count	:	3

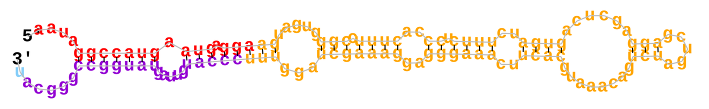

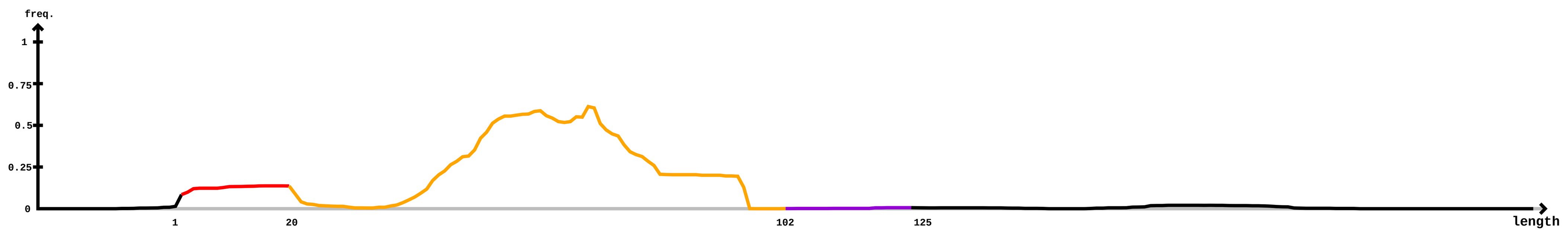

Mature Star

```
5'- cauuccggugcucaaucaaaaaaauaggccaugaaugaggaaguagugggccuuucacccucuuucuagugacucgaggagcugaucugacaaaugcacuucaaagggaggaaagcuagguuucccauguuaguauggccgggcauaaaagauuuugaaguuagguaaaaaauugggaaggaugaaguaggaucuaugaaauaucgaagaaaguuccagucaacuucaaauggaguuuucgauccagggaa -3' obs
    cauuccggugcucaaucaaaaaaauaggccaugaaugaggaaguagugggccuuucacccucuuucuagugacucgaggagcugaucugacaaaugcacuucaaagggaggaaagcuagguuucccauguuaguauggccgggcauaaaagauuuugaaguuagguaaaaaauugggaaggaugaaguaggaucuaugaaauaucgaagaaaguuccagucaacuucaaauggaguuuucgauccagggaa exp
    ..((((.((((((............(((((((.(((.(((((.....(((.((((..((.((((..((((......(((.....)))........))))..))))))..)))))))...))))))))).....))))))))))))).......((((((((.(......(((((((..((((..((((...))))....))))........)))))))))))))))))((((........)))))))).
```

alignment	reads	mm	sample
............caaucaaaaaaauaggccaugaaugagga	1	0	JCK-2
............caaucaaaaaaauaggccaugaaugagga	2	0	JCK-3
..............aucaaaaaaauaggccaugaaugaggaa	1	0	JT-2
...............ucaaaaaaauaggccaug	1	0	LT-3
...............ucaaaaaaauaggccaugaaugagg	1	0	JCK-1
...............ucaaaaaaauaggccaugaaugagg	1	0	JCK-3
...............ucaaaaaaauaggccaugaaugaggaa	1	0	JT-2
.................aaaaaaauaggccaugaaugagga	1	0	JCK-1
..................aaaaaauaggccaugaaugagga	1	0	LT-1
...................aaaaauaggccaugaaugagga	1	0	LT-1
...................aaaaauaggccaugaaugagga	3	0	JT-3
...................aaaaauaggccaugaaugagga	3	0	JT-2
....................aaaauaggccaugaaugagga	1	0	LT-1
.....................aaauaggccaugaaugagga	3	0	JT-3
.....................aaauaggccaugaaugagga	4	0	JT-2
.....................Caauaggccaugaaugaggaagua	1	1	JCK-3
.....................aaauaggccaugaaugaggaaguag	4	0	JT-3
......................aauaggccaugaaugagga	5	0	JT-2
......................aauaggccaugaaugagga	53	0	LCK-1
......................aauaggccaugaaNgagga	1	1	LCK-1
......................aauaggccaugaaugagga	42	0	LCK-2
......................aauaggccaugaaugagga	11	0	JT-3
......................aauaggccaugaaugaggaag	12	0	JT-2
......................aauaggccaugaaugaggaag	14	0	JT-3
......................aauaggccaugaaugaggaagu	4	0	LT-2
......................aauaggccaugaaugaggaagua	1	0	LT-3
......................aauaggccaugaaugaggaagua	4	0	LT-2
......................aauaggccaugaaugaggaaguagu	1	0	JCK-1
.......................auaggccaugaaugagga	5	0	JT-2
.......................auaggccaugaaugagga	1	0	LT-1
.......................auaggccaugaaugagga	15	0	JT-3
.......................auaggccaugaaugagga	1	0	JCK-1
.......................auaggccaugaaugaggaa	2	0	JT-3

```
                      Mature                                                                                              Star
cauuccggugcucaaucaaaaaaauaggccaugaaugaggaaguagugggccuuucacccucuuucuagugacucgaggagcugaucugacaaaugcacuucaaagggaggaaagcuagguuucccauguuaguauggccgggcauaaaagauuuugaaguuagguaaaaauugggaaggaugaaguaggaucuaugaaauaucgaagaaaguuccagucaacuucaaauggaguuucgauccagggaa
.......................auaggccaugaaugaggaa.......................................................................................................................................................................................................   1    0    JT-2
.......................auaggccaugCaugaggaa.......................................................................................................................................................................................................   1    1    JT-3
.......................auaggccaugaaugaggaaguagu..................................................................................................................................................................................................   1    0    JCK-2
.......................auaggccaugaaugaggaaguagu..................................................................................................................................................................................................   1    0    LT-3
.......................auaggccaugaaugaggaaguagug.................................................................................................................................................................................................   2    0    LT-2
........................uaggccaugaaugaggaa.......................................................................................................................................................................................................   17   0    LCK-1
........................uaggccaugaaugaggaa.......................................................................................................................................................................................................   20   0    LCK-2
........................uaggccaugaaugaggaa.......................................................................................................................................................................................................   3    0    JT-2
........................uaggccaugaaugaggaag......................................................................................................................................................................................................   1    0    LT-1
........................uaggccaugaaugaggaagu.....................................................................................................................................................................................................   2    0    LT-1
........................uaggccaugaaugaggaagua....................................................................................................................................................................................................   1    0    LT-1
........................uaggccaugaaugaggaagua....................................................................................................................................................................................................   1    0    JCK-1
.........................aggccaugaaugaggaagua....................................................................................................................................................................................................   5    0    LT-2
.........................aggccaugaCugaggaagua....................................................................................................................................................................................................   1    1    LT-2
.............................caugaaugaggaaguaguggg.......................................................................................................................................................................................................   1    0    JCK-1
.............................caugaaugaggaaguaguggg.......................................................................................................................................................................................................   2    0    JCK-2
.............................caugaaugaggaagguagugggc......................................................................................................................................................................................................   4    0    LCK-3
.............................caugaaugaggaaguagugggccuuuca................................................................................................................................................................................................   2    0    JT-3
..............................augaaugaggaaguagug.........................................................................................................................................................................................................   1    0    LT-3
..............................augaaugaggaaguaguggg.......................................................................................................................................................................................................   8    0    LCK-3
..............................augaaugaggaaguagugggc......................................................................................................................................................................................................   1    0    JCK-2
..............................augaaugaggaaguagugggc......................................................................................................................................................................................................   1    0    JCK-3
...............................ugaaugaggaaguagugggccuuucacccu............................................................................................................................................................................................   1    0    LT-1
................................gaaugaggaaguagugggc......................................................................................................................................................................................................   1    0    LT-1
.................................aaugaggaaguagugggc......................................................................................................................................................................................................   2    0    LT-2
.................................aaugaggaaguagugggccuuucac...............................................................................................................................................................................................   1    0    LT-3
..................................augaggaaguagugggccuuucacc..............................................................................................................................................................................................   1    0    LT-1
...................................ugaggaaguagugggccu....................................................................................................................................................................................................   2    0    LT-3
...................................ugaggaaguagugggccuuuc.................................................................................................................................................................................................   1    0    LT-1
...................................ugaggaaguagugggccuuucac...............................................................................................................................................................................................   1    0    LT-3
....................................gaggaaguagugggccuuucacccu............................................................................................................................................................................................   1    0    LT-1
.....................................................uucacccucuuucuagugacucgG............................................................................................................................................................................   1    1    LT-1
.......................................................cacccucuuucuagugacuc..............................................................................................................................................................................   8    0    LCK-3
.......................................................cacccucuuucuagugacucgG............................................................................................................................................................................   1    1    LT-1
........................................................acccucuuucuagugacucg.............................................................................................................................................................................   1    0    LT-3
.........................................................cccuUuuucuagugacuc..............................................................................................................................................................................   2    1    LT-3
.........................................................cccucuuucuagugacuc..............................................................................................................................................................................   1    0    LT-3
.........................................................cccucuuucuagugacucgGggagcu......................................................................................................................................................................   1    1    LT-3
.........................................................cccucuuucuagugacucgGggagcuga....................................................................................................................................................................   9    1    LCK-2
.........................................................cccucuuucuagugacucgGggagcuga....................................................................................................................................................................   6    1    LCK-1
..........................................................ccucuuucuagugacucg.............................................................................................................................................................................   3    0    LT-3
..........................................................ccucuuucuagugacucgGggagc.......................................................................................................................................................................   9    1    JT-1
..........................................................ccucuuucuagugacucgGggagcug.....................................................................................................................................................................   2    1    LT-1
...........................................................cucuuucuagugacucgGggagc.......................................................................................................................................................................   2    1    LT-1
...........................................................cucuuucuagugacucgGggagc.......................................................................................................................................................................   1    1    LT-3
...........................................................cucuuucuagugacucgGggagcu......................................................................................................................................................................   1    1    JCK-1
...........................................................cucuuucuagugacucgGggagcug.....................................................................................................................................................................   9    1    LCK-3
...........................................................cucuuucuagugacucgGggagcug.....................................................................................................................................................................   2    1    LT-1
...........................................................cucuuucuagugacucgGggagcug.....................................................................................................................................................................   3    1    LT-3
...........................................................cucuuucuagugacucgGggagcug.....................................................................................................................................................................   2    1    LT-2
...........................................................cucuuucuagugacucgGggagcugau...................................................................................................................................................................   5    1    LT-2
...........................................................cucuuucuagugacucgGggagcugauc..................................................................................................................................................................   2    1    LT-1
...........................................................cucuuucuagugacucgGggagcugauc..................................................................................................................................................................   1    1    LT-3
...........................................................cucuuucuagugacucgGggagcugauc..................................................................................................................................................................   1    1    JCK-3
...........................................................cucuuucuagugacucgGggagcugaucu.................................................................................................................................................................   1    1    JCK-3
............................................................ucuuucuagugacucgGggagc.......................................................................................................................................................................   2    1    LT-1
............................................................ucuuucuagugacucgGggagc.......................................................................................................................................................................   3    1    LT-3
............................................................ucuuucuagugacucgGggagc.......................................................................................................................................................................   6    1    JT-2
............................................................ucuuucuagugacucgGggagc.......................................................................................................................................................................   5    1    JT-3
............................................................ucuuucuagugacucgGggagc.......................................................................................................................................................................   5    1    LCK-3
............................................................ucuuucuagugacucgGggagcu......................................................................................................................................................................   1    1    LT-2
............................................................ucuuucuagugacucgGggagcu......................................................................................................................................................................   1    1    JCK-3
............................................................ucuuucuagugacucgGggagcugau...................................................................................................................................................................   1    1    LT-1
............................................................ucuuucuagugacucgGggagcugauc..................................................................................................................................................................   2    1    LT-1
............................................................ucuuucuagugacucgGggagcugaucu.................................................................................................................................................................   1    1    LT-1
............................................................ucuuucuagugacucgGggagcugaucug................................................................................................................................................................   2    1    JCK-2
............................................................ucuuucuagugacucgGggagcugaucug................................................................................................................................................................   3    1    JCK-1
............................................................ucuuucuagugacucgGggagcugaucug................................................................................................................................................................   3    1    JCK-3
.............................................................cuuucuagugacucgGggagc.......................................................................................................................................................................   4    1    LT-2
.............................................................cuuucuagugacucgGggagc.......................................................................................................................................................................   2    1    LT-3
```

Mature　　　　Star

cauuccggugcucaaucaaaaaaauaggccaugaaugaggaaguagugggccuuucacccucuuucuagugacucgaggagcugaucugacaaaugcacuucaaagggaggaaagcuagguuucccauguuaguauggccgggcauaaaagauuuugaaguuagguaaaaaauugggaaggaugaaguaggaucuaugaaauaucgaagaaaguuccagucaacuucaaauggaguuuucgauccagggaa

cuuucuagugacucgGggagc	4	1	LCK-3
cuuucuagugacucgGggagc	3	1	LT-1
cuuucuagugacucgGggagcu	1	1	JCK-1
cuuucuagugacucgGggagcu	4	1	JCK-3
cuuucuagugacucgGggagcu	1	1	LT-3
cuuucuagugacucgGggagcu	1	1	LT-1
cuuucuagugacucgGggagcug	1	1	LT-1
cuuucuagugacucgGggagcug	1	1	LT-3
cuuucuagugacucgGggagcuga	3	1	LT-2
cuuucuagugacucgGggagcuga	1	1	LT-1
cuuucuagugacucgGggagcugau	3	1	LT-2
cuuucuagugacucgGggagcugaucug	2	1	JCK-2
cuuucuagugacucgGggagcugaucug	1	1	JCK-3
cuuucuagugacucgGggagcugaucug	2	1	JCK-1
cuuucuagugacucgGggagcugaucuga	2	1	JCK-1
cuuucuagugacucgGggagcugaucuga	3	1	JCK-2
uuucuagugacucgGggag	1	1	LT-3
uuucuagugacucgGggagc	1	1	LT-1
uuucuagugacucgGggagcu	6	1	JT-3
uuucuagugacucgGggagcu	1	1	LT-2
uuucuagugacucgGggagcu	1	1	LT-3
uuucuagugacucgGggagcu	1	1	JCK-2
uuucuagugacucgGggagcu	1	1	JCK-1
uuucuagugacucgGggagcu	1	1	JCK-3
uuucuagugacucgGggagcu	2	1	JT-2
uuucuagugacucgGggagcug	6	1	LCK-3
uuucuagugacucgGggagcug	2	1	LT-3
uuucuagugacucgGggagcuga	1	1	LT-1
uuucuagugacucgGggagcugau	3	1	LT-1
uuucuagugacucgGggagcugauc	1	1	LT-1
uuucuagugacucgGggagcugauc	1	1	JCK-2
uuucuagugacucgGggagcugauc	1	1	JCK-3
uuucuagugacucgGggagcugauc	5	1	LT-2
uuucuagugacucgGggagcugaucu	3	1	LT-2
uuucuagugacucgGggagcugaucuga	2	1	JCK-2
uuucuagugacucgGggagcugaucuga	3	1	LT-2
uuucuagugacucgGggagcugaucugac	1	1	JCK-1
uuucuagugacucgGggagcugaucugaca	1	1	LT-2
uuucuagugacucgGggagcugaucugaca	3	1	LCK-3
uucuagugacucgGggag	1	1	LCK-3
uucuagugacucgGggagc	8	1	LCK-3
uucuagugacucgGggagc	2	1	LT-1
uucuagugacucgGggagc	5	1	LT-3
uucuagugacucgGggagcu	2	1	JCK-2
uucuagugacucgGggagcu	1	1	LT-3
uucuagugacucgGggagcuga	6	1	JT-3
uucuagugacucgGggagcuga	2	1	JT-2
uucuagugacucgGggagcuga	1	1	LT-1
uucuagugacucgGggagcugau	1	1	LT-3
uucuagugacucgGggagcugaucug	6	1	JT-1
uucuagugacucgGggagcugaucug	1	1	LT-3
uucuagugacucgGggagcugaucuga	1	1	LCK-3
uucuagugacucgGggagcugaucugac	1	1	LT-1
uucuagugacucgGggagcugaucugaca	3	1	JCK-1
uucuagugacucgGggagcugaucugaca	1	1	LT-1
uucuagugacucgGggagcugaucugaca	3	1	JCK-3
uucuagugacucgGggagcugaucugaca	1	1	LT-3
uucuagugacucgGggagcugaucugaca	2	1	JCK-2
uucuagugacucgGggagcugaucugaca	2	1	LCK-3
uucuagugacucgGggagcugaucugacaa	1	1	JCK-3
uucuagugacucgGggagcugaucugacaa	1	1	LT-1
ucuagugacucgGggagc	1	1	LT-1
ucuagugacucgGggagc	1	1	JCK-1
ucuagugacucgGggagc	1	1	LT-3
ucuagugacucgGggagc	1	1	LT-2
ucuagugacucgGggagcu	4	1	LCK-3
ucuagugacucgGggagcu	2	1	LT-1
ucuagugacucgGggagcu	1	1	JCK-3
ucuagugacucgGggagcu	11	1	LT-2
ucuagugacucgGggagcu	2	1	JCK-2

```
                      Mature                                                                                               Star
cauuccggugcucaaucaaaaaaauaggccaugaaugaggaaguagugggccuuucacccucuuucuagugacucgaggagcugaucugacaaaugcacuucaaagggaggaaagcuagguuucccauguuaguauggccgggcauaaaagauuuugaaguuagguaaaaaauugggaaggaugaaguaggaucuaugaaauaucgaagaaaguuccagucaacuucaaauggaguuucgauccagggaa
................................................................ucuagugacucgGggagcu.......................................................................................................................................................................   2   1   JCK-1
................................................................ucuagugacucgGggagcug......................................................................................................................................................................   2   1   LT-3
................................................................ucuagugacucgGggagcuga.....................................................................................................................................................................   8   1   JT-1
................................................................ucuagugacucgGggagcugau....................................................................................................................................................................   1   1   LT-3
................................................................ucuagugacucgGggagcugau....................................................................................................................................................................   1   1   LT-1
................................................................ucuagugacucgGggagcugau....................................................................................................................................................................   1   1   LT-2
................................................................ucuagugacucgGggagcugauc...................................................................................................................................................................   4   1   LT-3
................................................................ucuagugacucgGggagcugauc...................................................................................................................................................................   3   1   LT-1
................................................................ucuagugacucgGggagcugaucu..................................................................................................................................................................   2   1   JCK-2
................................................................ucuagugacucgGggagcugaucu..................................................................................................................................................................   1   1   LT-3
................................................................ucuagugacucgGggagcugaucu..................................................................................................................................................................   18  1   LCK-2
................................................................ucuagugacucgGggagcugaucu..................................................................................................................................................................   1   1   JCK-1
................................................................ucuagugacucgGggagcugaucu..................................................................................................................................................................   2   1   LT-1
................................................................ucuagugacucgGggagcugaucu..................................................................................................................................................................   15  1   LCK-1
................................................................ucuagugacucgGggagcugaucug.................................................................................................................................................................   2   1   LT-1
................................................................ucuagugacucgGggagcugaucug.................................................................................................................................................................   4   1   LT-3
................................................................ucuagugacucgGggagcugaucuga................................................................................................................................................................   1   1   JCK-3
................................................................ucuagugacucgGggagcugaucuga................................................................................................................................................................   1   1   JCK-1
................................................................ucuagugacucgGggagcugaucuga................................................................................................................................................................   1   1   LT-2
................................................................ucuagugacucgGggagcugaucugaca..............................................................................................................................................................   2   1   LT-1
................................................................ucuagugacucgGggagcugaucugaca..............................................................................................................................................................   1   1   JT-3
................................................................ucuagugacucgGggagcugaucugaca..............................................................................................................................................................   7   1   LCK-3
................................................................ucuagugacucgGggagcugaucugacaa.............................................................................................................................................................   2   1   JCK-2
................................................................ucuagugacucgGggagcugaucugacaa.............................................................................................................................................................   2   1   JCK-1
................................................................ucuagugacucgGggagcugaucugacaa.............................................................................................................................................................   1   1   LT-2
.................................................................cuagugacucgGggagcu.......................................................................................................................................................................   2   1   JCK-2
.................................................................cuagugacucgGggagcu.......................................................................................................................................................................   1   1   JCK-3
.................................................................cuagugacucgGggagcu.......................................................................................................................................................................   1   1   LT-3
.................................................................cuagugacucgGggagcu.......................................................................................................................................................................   11  1   LT-2
.................................................................cuagugacucgGggagcu.......................................................................................................................................................................   2   1   LT-1
.................................................................cuagugacucgGggagcug......................................................................................................................................................................   3   1   LT-1
.................................................................cuagugacucgGggagcug......................................................................................................................................................................   1   1   LT-2
.................................................................cuagugacucgGggagcug......................................................................................................................................................................   1   1   LT-3
.................................................................cuagugacucgGggagcuga.....................................................................................................................................................................   1   1   JCK-2
.................................................................cuagugacucgGggagcuga.....................................................................................................................................................................   2   1   JCK-3
.................................................................cuagugacucgGggagcugau....................................................................................................................................................................   1   1   LCK-3
.................................................................cuagugacucgGggagcugau....................................................................................................................................................................   1   1   LT-1
.................................................................cuagugacucgGggagcugauc...................................................................................................................................................................   1   1   LT-1
.................................................................cuagugacucgGggagcugaucu..................................................................................................................................................................   1   1   JCK-1
.................................................................cuagugacucgGggagcugaucu..................................................................................................................................................................   1   1   LT-1
.................................................................cuagugacucgGggagcugaucu..................................................................................................................................................................   1   1   LT-3
.................................................................cuagugacucgGggagcugaucu..................................................................................................................................................................   2   1   LT-2
.................................................................cuagugacucgGggagcugaucu..................................................................................................................................................................   4   1   JCK-2
.................................................................cuagugacucgGggagcugaucu..................................................................................................................................................................   1   1   JCK-3
.................................................................cuagugacucgGggagcugaucug.................................................................................................................................................................   2   1   JCK-2
.................................................................cuagugacucgGggagcugaucug.................................................................................................................................................................   5   1   LT-3
.................................................................cuagugacucgGggagcugaucuga................................................................................................................................................................   2   1   JT-1
.................................................................cuagugacucgGggagcugaucugac...............................................................................................................................................................   1   1   LT-3
.................................................................cuagugacucgGggagcugaucugaca..............................................................................................................................................................   1   1   JCK-3
.................................................................cuagugacucgGggagcugaucugaca..............................................................................................................................................................   3   1   LT-1
.................................................................cuagugacucgGggagcugaucugaca..............................................................................................................................................................   5   1   JCK-1
.................................................................cuagugacucgGggagcugaucugaca..............................................................................................................................................................   1   1   JCK-2
.................................................................cuagugacucgGggagcugaucugacaa.............................................................................................................................................................   1   1   LT-2
.................................................................cuagugacucgGggagcugaucugacaaa............................................................................................................................................................   5   1   JCK-3
.................................................................cuagugacucgGggagcugaucugacaaa............................................................................................................................................................   4   1   JCK-2
.................................................................cuagugacucgGggagcugaucugacaaau...........................................................................................................................................................   1   1   LT-2
.................................................................cuagugacucgGggagcugaucugacaaau...........................................................................................................................................................   2   1   LCK-3
..................................................................uagugacucgGggagcug......................................................................................................................................................................   1   1   LT-3
..................................................................uagugacucgGggagcug......................................................................................................................................................................   2   1   LT-2
..................................................................uagugacucgGggagcuga.....................................................................................................................................................................   1   1   LT-3
..................................................................uagugacucgGggagcugau....................................................................................................................................................................   2   1   LT-2
..................................................................uagugacucgGggagcugauc...................................................................................................................................................................   1   1   LT-1
..................................................................uagugacucgGggagcugauc...................................................................................................................................................................   3   1   LT-2
..................................................................uagugacucgGggagcugauc...................................................................................................................................................................   3   1   LT-3
..................................................................uagugacucgGggagcugaucu..................................................................................................................................................................   4   1   LCK-3
..................................................................uagugacucgGggagcugaucu..................................................................................................................................................................   2   1   LT-1
..................................................................uagugacucgGggagcugaucug.................................................................................................................................................................   1   1   JCK-1
..................................................................uagugacucgGggagcugaucug.................................................................................................................................................................   1   1   LT-1
..................................................................uagugacucgGggagcugaucug.................................................................................................................................................................   1   1   LT-2
..................................................................uagugacucgGggagcugaucugac...............................................................................................................................................................   1   1   JCK-3
```

Mature · **Star**

cauuccggugcucaaucaaaaa**aauaggccaugaaugagga**aguagugggccuuucacccucuuucuagugacucgaggagcugaucugacaaaugcacuucaaagggaggaaagcuagguuu**cccauguuaguauggccgggca**uaaaagauuuugaaguuagguaaaaauugggaaggaugaaguaggaucuaugaaauaucgaagaaaguuccagucaacuucaaauggaguuuucgauccagggaa

uagugacucgGggagcugaucugac	1	1	JCK-1
uagugacucgGggagcugaucugac	1	1	LT-3
uagugacucgaggagcugaucugac	1	0	JT-1
uagugacucgGggagcugaucugac	1	1	JCK-2
uagugacucgGggagcugaucugaca	1	1	LT-3
uagugacucgGggagcugaucugacaa	1	1	JCK-3
uagugacucgGggagcugaucugacaa	2	1	LCK-2
uagugacucgGggagcugaucugacaa	1	1	LT-1
uagugacucgGggagcugaucugacaa	5	1	LT-2
uagugacucgGggagcugaucugacaa	2	1	JCK-1
uagugacucgGggagcugaucugacaa	4	1	JCK-2
uagugacucgGggagcugaucugacaaa	1	1	LT-3
uagugacucgGggagcugaucugacaaa	1	1	LT-2
uagugacucgGggagcugaucugacaaau	1	1	LCK-3
uagugacucgGggagcugaucugacaaaug	2	1	LCK-3
uagugacucgGggagcugaucugacaaaug	2	1	LT-1
agugacucgGggagcuga	4	1	JT-3
agugacucgGggagcuga	1	1	LT-3
agugacucgGggagcuga	2	1	JT-2
agugacucgGggagcugauc	4	1	LT-2
agugacucgGggagcugaucu	1	1	LT-1
agugacucgGggagcugaucu	6	1	JT-3
agugacucgGggagcugaucu	1	1	JT-2
agugacucgGggagcugaucu	2	1	LT-3
agugacucgGggagcugaucug	9	1	JT-1
agugacucgGggagcugaucug	3	1	LT-2
agugacucgGggagcugaucugac	2	1	LT-3
agugacucgGggagcugaucugac	1	1	LT-1
agugacucgGggagcugaucugaca	2	1	JCK-1
agugacucgGggagcugaucugaca	1	1	LT-3
agugacucgGggagcugaucugaca	1	1	JCK-3
agugacucgGggagcugaucugaca	1	1	LT-1
agugacucgGggagcugaucugaca	4	1	LT-2
agugacucgGggagcugaucugaca	5	1	JCK-2
agugacucgGggagcugaucugacaa	1	1	JCK-1
agugacucgGggagcugaucugacaa	1	1	JCK-3
agugacucgGggagcugaucugacaa	2	1	JCK-2
agugacucgGggagcugaucugacaa	3	1	LT-3
agugacucgGggagcugaucugacaaa	1	1	JCK-2
agugacucgGggagcugaucugacaaa	1	1	JCK-1
agugacucgGggagcugaucugacaaau	2	1	LT-1
agugacucgGggagcugaucugacaaaug	7	1	LCK-1
agugacucgGggagcugaucugacaaaug	4	1	LCK-2
agugacucgGggagcugaucugacaaaug	2	1	LT-1
agugacucgGggagcugaucugacaaaug	3	1	LT-2
agugacucgGggagcugaucugacaaaugc	1	1	LT-3
gugacucgGggagcugauc	1	1	JCK-2
gugacucgGggagcugaucug	2	1	JCK-2
gugacucgGggagcugaucug	2	1	LT-1
gugacucgGggagcugaucuga	6	1	LT-2
gugacucgGggagcugaucugaca	1	1	LT-1
gugacucgGggagcugaucugaca	11	1	JT-1
gugacucgGggagcugaucugaca	3	1	JT-2
gugacucgGggagcugaucugaca	1	1	JCK-1
gugacucgGggagcugaucugaca	5	1	JT-3
gugacucgGggagcugaucugacaa	3	1	JT-3
gugacucgGggagcugaucugacaa	2	1	JT-2
gugacucgGggagcugaucugacaa	1	1	LT-3
gugacucgGggagcugaucugacaaa	1	1	JCK-3
gugacucgGggagcugaucugacaaau	4	1	JCK-2
ugacucgGggagcugauc	1	1	JCK-2
ugacucgGggagcugauc	5	1	LCK-3
ugacucgGggagcugauc	1	1	LT-1
ugacucgGggagcugauc	1	1	JCK-3
ugacucgGggagcugaucu	1	1	LT-3
ugacucgGggagcugaucugac	2	1	LT-1
ugacucgGggagcugaucugaca	2	1	LT-3
ugacucgGggagcugaucugaca	8	1	LT-2
ugacucgGggagcugaucugaca	3	1	LT-1
ugacucgGggagcugaucugaca	12	1	LCK-3

Mature **Star**

cauuccggugcucaaucaaaaaaauaggccaugaaugaggaaguagugggccuuucacccucuuucuagugacucgaggagcugaucugacaaaugcacuucaaagggaggaaagcuagguuucccauguuaguauggccgggcauaaaagauuuugaaguuagguaaaaaauugggaaggaugaaguaggaucuaugaaauaucgaagaaaguuccagucaacuucaaauggaguuucgauccagggaa

ugacucgGggagcugaucugacaa	1	1	LT-2
ugacucgGggagcugaucugacaa	1	1	JCK-3
ugacucgGggagcugaucugacaa	1	1	JCK-2
ugacucgGggagcugaucugacaa	2	1	JCK-1
ugacucgGggagcugaucugacaa	3	1	LT-1
ugacucgGggagcugaucugacaaau	1	1	LT-2
ugacucgGggagcugaucugacaaaug	3	1	LT-2
ugacucgGggagcugaucugacaaaug	1	1	LT-3
ugacucgGggagcugaucugacaaaugc	1	1	JCK-2
ugacucgGggagcugaucugacaaaugc	1	1	LT-1
ugacucgGggagcugaucugacaaaugc	1	1	JCK-1
ugacucgGggagcugaucugacaaaugc	1	1	LT-2
ugacucgGggagcugaucugacaaaugca	3	1	LCK-3
ugacucgGggagcugaucugacaaaugcac	2	1	JCK-3
gacucgGggagcugaucugacaaa	1	1	JCK-3
gacucgGggagcugaucugacaaa	2	1	LT-2
gacucgGggagcugaucugacaaaug	1	1	LT-3
gacucgGggagcugaucugacaaaugc	2	1	LT-3
gacucgGggagcugaucugacaaaugc	3	1	JCK-1
acucgGggagcugaucuga	1	1	LT-3
acucgGggagcugaucugac	4	1	LT-2
acucgGggagcugaucugaca	6	1	JCK-2
acucgGggagcugaucugaca	5	1	LT-3
acucgGggagcugaucugaca	1	1	LT-1
acucgGggagcugaucugaca	1	1	JCK-1
acucgGggagcugaucugaca	1	1	JCK-3
acucgGggagcugaucugaca	5	1	LT-2
acucgGggagcugaucugacaa	2	1	LT-3
acucgGggagcugaucugacaa	2	1	LT-1
acucgGggagcugaucugacaa	1	1	JCK-3
acucgGggagcugaucugacaa	9	1	LT-2
acucgGggagcugaucugacaaa	2	1	LT-1
acucgGggagcugaucugacaaau	1	1	LT-1
acucgGggagcugaucugacaaau	3	1	LT-2
acucgGggagcugaucugacaaaug	2	1	LT-1
acucgGggagcugaucugacaaaug	4	1	LT-3
acucgGggagcugaucugacaaaug	2	1	JCK-2
acucgGggagcugaucugacaaaug	2	1	JCK-3
acucgGggagcugaucugacaaaug	1	1	JCK-1
acucgGggagcugaucugacaaaug	3	1	JT-3
acucgGggagcugaucugacaaaug	3	1	JT-2
acucgGggagcugaucugacaaaug	2	1	LT-2
acucgGggagcugaucugacaaaugc	2	1	LT-2
acucgGggagcugaucugacaaaugc	1	1	JCK-1
acucgGggagcugaucugacaaaugc	3	1	LT-1
acucgGggagcugaucugacaaaugc	1	1	JCK-2
acucgGggagcugaucugacaaaugca	1	1	JCK-1
acucgGggagcugaucugacaaaugca	2	1	JCK-2
acucgGggagcugaucugacaaaugcacu	1	1	LT-1
acucgGggagcugaucugacaaaugcacu	3	1	JCK-2
cucgGggagcugaucuga	7	1	JT-2
cucgGggagcugaucuga	9	1	JT-3
cucgGggagcugaucuga	4	1	LT-3
cucgGggagcugaucugac	1	1	LT-1
cucgGggagcugaucugaca	29	1	LCK-2
cucgGggagcugaucugaca	4	1	JCK-1
cucgGggagcugaucugaca	4	1	LT-3
cucgGggagcugaucugaca	2	1	JCK-3
cucgGggagcugaucugaca	1	1	LT-1
cucgGggagcugaucugaca	1	1	JCK-2
cucgGggagcugaucugaca	15	1	LCK-1
cucgGggagcugaucugacaa	1	1	LT-3
cucgGggagcugaucugacaa	5	1	LCK-3
cucgGggagcugaucugacaa	4	1	JCK-2
cucgGggagcugaucugacaa	1	1	LT-1
cucgGggagcugaucugacaa	1	1	JCK-3
cucgGggagcugaucugacaa	2	1	LT-2
cucgGggagcugaucugacaaa	6	1	LT-2
cucgGggagcugaucugacaaa	1	1	LT-3
cucgGggagcugaucugacaaa	1	1	LT-1

Mature   Star

cauuccggugcucaaucaaaaaaauaggccaugaaugaggaaguagugggccuuucacccucuuucuagugacucgaggagcugaucugacaaaugcacuucaaagggaggaaagcuagguuucccauguuaguauggccgggcauaaaagauuuugaaguuagguaaaaauugggaaggaugaaguaggaucuaugaaauaucgaagaaaguuccagucaacuucaaauggaguuuucgauccagggaa

cucgGggagcugaucugacaaau	1	1	LT-1
cucgGggagcugaucugacaaau	1	1	LT-3
cucgGggagcugaucugacaaaug	4	1	JT-1
cucgGggagcugaucugacaaaug	2	1	LT-1
cucgGggagcugaucugacaaaugc	1	1	JCK-3
cucgGggagcugaucugacaaaugc	1	1	JCK-1
cucgGggagcugaucugacaaaugc	5	1	LT-2
cucgGggagcugaucugacaaaugc	3	1	LT-1
cucgGggagcugaucugacaaaugc	3	1	JT-3
cucgGggagcugaucugacaaaugc	3	1	JCK-2
cucgGggagcugaucugacaaaugca	3	1	LT-2
cucgGggagcugaucugacaaaugca	3	1	JCK-1
cucgGggagcugaucugacaaaugca	8	1	JT-1
cucgGggagcugaucugacaaaugca	1	1	LT-1
cucgGggagcugaucugacaaaugcac	1	1	JCK-2
cucgGggagcugaucugacaaaugcac	2	1	JCK-1
cucgGggagcugaucugacaaaugcacu	1	1	JCK-3
cucgGggagcugaucugacaaaugcacu	1	1	LT-2
cucgGggagcugaucugacaaaugcacu	1	1	LT-3
cucgGggagcugaucugacaaaugcacu	1	1	JCK-2
cucgGggagcugaucugacaaaugcacu	1	1	LT-1
cucgGggagcugaucugacaaaugcacu	1	1	JCK-1
cucgGggagcugaucugacaaaugcacuuc	1	1	LT-2
cucgGggagcugaucugacaaaugcacuuc	1	1	JCK-3
ucgGggagcugaucugaca	8	1	LCK-1
ucgGggagcugaucugaca	2	1	LT-3
ucgGggagcugaucugaca	7	1	LCK-2
ucgGggagcugaucugaca	1	1	LT-1
ucgGggagcugaucugacaa	1	1	LT-1
ucgGggagcugaucugacaa	5	1	LT-2
ucgGggagcugaucugacaaa	3	1	LT-1
ucgGggagcugaucugacaaa	5	1	LT-2
ucgGggagcugaucugacaaa	1	1	JCK-2
ucgGggagcugaucugacaaau	4	1	LT-2
ucgGggagcugaucugacaaau	1	1	JCK-2
ucgGggagcugaucugacaaau	1	1	LT-1
ucgGggagcugaucugacaaaug	2	1	LT-3
ucgGggagcugaucugacaaaug	2	1	JCK-2
ucgGggagcugaucugacaaaug	2	1	LT-1
ucgGggagcugaucugacaaaugc	2	1	LT-3
ucgGggagcugaucugacaaaugc	5	1	LT-2
ucgGggagcugaucugacaaaugca	1	1	LT-1
ucgGggagcugaucugacaaaugca	3	1	LT-3
ucgGggagcugaucugacaaaugca	4	1	JCK-2
ucgGggagcugaucugacaaaugca	1	1	JCK-1
ucgGggagcugaucugacaaaugca	2	1	JCK-3
ucgGggagcugaucugacaaaugcac	2	1	LT-3
ucgGggagcugaucugacaaaugcacu	2	1	JCK-3
ucgGggagcugaucugacaaaugcacu	1	1	LT-1
ucgGggagcugaucugacaaaugcacu	1	1	JCK-2
ucgGggagcugaucugacaaaugcacuu	2	1	LT-1
ucgGggagcugaucugacaaaugcacuuc	2	1	LT-2
ucgGggagcugaucugacaaaugcacuuca	2	1	LT-2
cgGggagcugaucugaca	3	1	LCK-3
cgGggagcugaucugaca	2	1	LT-1
cgGggagcugaucugaca	1	1	LT-3
cgGggagcugaucugacaa	1	1	LT-3
cgGggagcugaucugacaa	3	1	LT-2
cgGggagcugaucugacaaa	1	1	LT-3
cgGggagcugaucugacaaa	5	1	LT-2
cgGggagcugaucugacaaau	1	1	LT-3
cgGggagcugaucugacaaaug	3	1	LT-3
cgGggagcugaucugacaaaug	3	1	JCK-1
cgGggagcugaucugacaaaug	2	1	LT-1
cgGggagcugaucugacaaaug	4	1	LT-2
cgGggagcugaucugacaaaugc	3	1	LT-3
cgGggagcugaucugacaaaugc	4	1	JCK-2
cgGggagcugaucugacaaaugc	2	1	LT-2
cgGggagcugaucugacaaaugc	8	1	LCK-3
cgGggagcugaucugacaaaugcac	1	1	LT-1

```
                      Mature                                                                                               Star
cauuccggugcucaaucaaaaaaauaggccaugaaugaggaaguagugggccuuucacccucuuucuagugacucgaggagcugaucugacaaaugcacuucaaagggaggaaagcuagguuucccauguuaguauggccgggcauaaaagauuuugaaguuagguaaaaauugggaaggaugaaguaggaucuaugaaauaucgaagaaaguuccagucaacuucaaauggaguuucgauccagggaa
..........................................................................cgGgagcugaucugacaaaugcacu.......................................................................................................................................................   2   1   JCK-1
..........................................................................cgGgagcugaucugacaaaugcacu.......................................................................................................................................................   1   1   LT-1
..........................................................................cgGgagcugaucugacaaaugcacu.......................................................................................................................................................   7   1   JT-1
..........................................................................cgGgagcugaucugacaaaugcacu.......................................................................................................................................................   2   1   JCK-3
..........................................................................cgGgagcugaucugacaaaugcacu.......................................................................................................................................................   4   1   JCK-2
..........................................................................cgGgagcugaucugacaaaugcacu.......................................................................................................................................................   2   1   LT-3
..........................................................................cgGgagcugaucugacaaaugcacuu......................................................................................................................................................   6   1   LCK-1
..........................................................................cgGgagcugaucugacaaaugcacuu......................................................................................................................................................   1   1   JT-3
..........................................................................cgGgagcugaucugacaaaugcacuu......................................................................................................................................................   1   1   LT-2
..........................................................................cgGgagcugaucugacaaaugcacuu......................................................................................................................................................   20  1   LCK-2
..........................................................................cgGgagcugaucugacaaaugcacuu......................................................................................................................................................   1   1   JT-2
..........................................................................cgGgagcugaucugacaaaugcacuu......................................................................................................................................................   1   1   LT-3
..........................................................................cgGgagcugaucugacaaaugcacuuc.....................................................................................................................................................   6   1   LCK-1
..........................................................................cgGgagcugaucugacaaaugcacuuc.....................................................................................................................................................   11  1   LCK-2
..........................................................................cgGgagcugaucugacaaaugcacuuca....................................................................................................................................................   1   1   LT-1
..........................................................................cgGgagcugaucugacaaaugcacuucaa...................................................................................................................................................   1   1   LT-1
...........................................................................gGgagcugaucugacaaa.............................................................................................................................................................   4   1   LT-2
...........................................................................gGgagcugaucugacaaa.............................................................................................................................................................   2   1   LT-3
...........................................................................gGgagcugaucugacaaau............................................................................................................................................................   1   1   LT-1
...........................................................................gGgagcugaucugacaaaug...........................................................................................................................................................   3   1   LT-2
...........................................................................gGgagcugaucugacaaaug...........................................................................................................................................................   3   1   JCK-3
...........................................................................gGgagcugaucugacaaaug...........................................................................................................................................................   4   1   LT-1
...........................................................................gGgagcugaucugacaaaug...........................................................................................................................................................   2   1   LT-3
...........................................................................gGgagcugaucugacaaaugc..........................................................................................................................................................   4   1   LT-3
...........................................................................gGgagcugaucugacaaaugc..........................................................................................................................................................   4   1   LT-2
...........................................................................gGgagcugaucugacaaaugc..........................................................................................................................................................   8   1   LT-1
...........................................................................gGgagcugaucugacaaaugc..........................................................................................................................................................   1   1   JCK-2
...........................................................................gGgagcugaucugacaaaugca.........................................................................................................................................................   3   1   JCK-2
...........................................................................gGgagcugaucugacaaaugca.........................................................................................................................................................   1   1   LT-1
...........................................................................gGgagcugaucugacaaaugcac........................................................................................................................................................   1   1   LT-1
...........................................................................gGgagcugaucugacaaaugcac........................................................................................................................................................   1   1   JCK-1
...........................................................................gGgagcugaucugacaaaugcacu.......................................................................................................................................................   4   1   LT-2
...........................................................................gGgagcugaucugacaaaugcacu.......................................................................................................................................................   2   1   JCK-1
...........................................................................gGgagcugaucugacaaaugcacu.......................................................................................................................................................   3   1   LCK-3
...........................................................................gGgagcugaucugacaaaugcacu.......................................................................................................................................................   2   1   LT-1
...........................................................................gGgagcugaucugacaaaugcacu.......................................................................................................................................................   7   1   JCK-3
...........................................................................gGgagcugaucugacaaaugcacuu......................................................................................................................................................   2   1   LCK-3
...........................................................................gGgagcugaucugacaaaugcacuu......................................................................................................................................................   2   1   LT-1
............................................................................Gggagcugaucugacaaa.............................................................................................................................................................   2   1   LT-3
............................................................................Gggagcugaucugacaaau............................................................................................................................................................   1   1   LT-3
............................................................................Gggagcugaucugacaaaug...........................................................................................................................................................   14  1   LCK-1
............................................................................Gggagcugaucugacaaaug...........................................................................................................................................................   10  1   LCK-2
............................................................................Gggagcugaucugacaaaug...........................................................................................................................................................   1   1   JCK-2
............................................................................Gggagcugaucugacaaaugc..........................................................................................................................................................   1   1   LT-3
............................................................................Gggagcugaucugacaaaugc..........................................................................................................................................................   1   1   LT-1
............................................................................Gggagcugaucugacaaaugc..........................................................................................................................................................   3   1   LT-2
............................................................................Gggagcugaucugacaaaugcac........................................................................................................................................................   1   1   JCK-2
............................................................................Gggagcugaucugacaaaugcac........................................................................................................................................................   1   1   JCK-3
............................................................................Gggagcugaucugacaaaugcac........................................................................................................................................................   2   1   LT-2
............................................................................Gggagcugaucugacaaaugcac........................................................................................................................................................   1   1   JCK-1
............................................................................Gggagcugaucugacaaaugcacu.......................................................................................................................................................   1   1   LT-1
............................................................................Gggagcugaucugacaaaugcacuu......................................................................................................................................................   2   1   LT-1
.............................................................................ggagcugaucugacaaaug..........................................................................................................................................................   1   0   JCK-3
.............................................................................ggagcugaucugacaaaug..........................................................................................................................................................   1   0   JCK-1
..............................................................................gagcugaucugacaaaug..........................................................................................................................................................   3   0   LT-2
..............................................................................gagcugaucugacaaaug..........................................................................................................................................................   3   0   LT-3
..............................................................................gagcugaucugacaaaugc.........................................................................................................................................................   2   0   LT-3
..............................................................................gagcugaucugacaaaugc.........................................................................................................................................................   4   0   LCK-3
...............................................................................agcugaucugacaaaugc.........................................................................................................................................................   2   0   LT-3
...............................................................................agcugaucugacaaaugcac.......................................................................................................................................................   1   0   JCK-3
...............................................................................agcugaucugacaaaugcac.......................................................................................................................................................   2   0   JCK-2
...............................................................................agcugaucugacaaaugcacC......................................................................................................................................................   1   1   JCK-2
...............................................................................agcugaucugacaaaugcacuu.....................................................................................................................................................   2   0   LT-2
...............................................................................agcugaucugacaaaugcacuu.....................................................................................................................................................   2   0   JCK-2
...............................................................................agcugaucugacaaaugcacuu.....................................................................................................................................................   1   0   LT-3
................................................................................gcugaucugacaaaugcacuu.....................................................................................................................................................   2   0   LT-3
................................................................................gcugaucugacaaaugcacuucaaaggga.............................................................................................................................................   1   0   LT-2
.................................................................................cugaucugacaaaugcac.......................................................................................................................................................   1   0   JCK-1
.................................................................................cugaucugacaaaugcac.......................................................................................................................................................   1   0   LT-1
.................................................................................cugaucugacaaaugcac.......................................................................................................................................................   3   0   LT-2
```

```
                      Mature                                                                                                    Star
cauuccggugcucaaucaaaaaaauaggccaugaaugaggaaguagugggccuuucacccucuuucuagugacucgaggagcugaucugacaaaugcacuucaaagggaggaaagcuagguuucccauguuaguauggccgggcauaaaagauuuugaaguuagguaaaaauugggaaggaugaaguaggaucuaugaaauaucgaagaaaguuccagucaacuucaaauggaguuuucgauccagggaa
.................................................................................cugaucugacaaaugcac.......................................................................................................................................................   1   0   JCK-2
.................................................................................cugaucugacaaaugcacu......................................................................................................................................................   2   0   LT-2
.................................................................................cugaucugacaaaugcacu......................................................................................................................................................   2   0   LT-1
.................................................................................cugaucugacaaaugcacuu.....................................................................................................................................................   2   0   LT-3
.................................................................................cugaucugacaaaugcacuu.....................................................................................................................................................   2   0   LT-1
.................................................................................cugaucugacaaaugcacuuc....................................................................................................................................................   1   0   JCK-1
.................................................................................cugaucugacaaaugcacuuc....................................................................................................................................................   2   0   LT-3
.................................................................................cugaucugacaaaugcacuuc....................................................................................................................................................   10  0   LT-2
.................................................................................cugaucugCcaaaugcacuuc....................................................................................................................................................   1   1   LT-2
.................................................................................cugaucugacaaaugcacuuc....................................................................................................................................................   3   0   JCK-2
.................................................................................cugaucugacaaaugcacuuc....................................................................................................................................................   4   0   JCK-3
..................................................................................ugaucugacaaaugcacu......................................................................................................................................................   1   0   LT-3
..................................................................................ugaucugacaaaugcacu......................................................................................................................................................   3   0   LT-1
..................................................................................ugaucugacaaaugcacuu.....................................................................................................................................................   2   0   LT-1
..................................................................................ugaucugacaaaugcacuu.....................................................................................................................................................   1   0   JCK-2
..................................................................................ugaucugCcaaaugcacuuc....................................................................................................................................................   1   1   LCK-2
..................................................................................ugaucugacaaaugcacuuc....................................................................................................................................................   7   0   LT-1
..................................................................................ugaucugacaaaugcacuuc....................................................................................................................................................   33  0   LCK-2
..................................................................................ugaucugacaaaugcacuuc....................................................................................................................................................   2   0   LT-3
..................................................................................ugaucugacaaaugcacuuc....................................................................................................................................................   19  0   LCK-1
..................................................................................ugaucugacaaaugcacuucaaaggga.............................................................................................................................................   2   0   JCK-3
..................................................................................ugaucugacaaaugcacuucaaagggG.............................................................................................................................................   1   1   JCK-2
..................................................................................ugaucugacaaaugcacuucaaaggga.............................................................................................................................................   1   0   JCK-1
..................................................................................ugaucugacaaaugcacuucaaaggga.............................................................................................................................................   2   0   JCK-2
...................................................................................gaucugacaaaugcacuu.....................................................................................................................................................   1   0   LT-2
...................................................................................gaucugacaaaugcacuu.....................................................................................................................................................   1   0   LT-3
...................................................................................gaucugacaaaugcacuuc....................................................................................................................................................   1   0   JCK-2
....................................................................................aucugacaaaugcacuuc....................................................................................................................................................   2   0   JCK-2
....................................................................................aucugacaaaugcacuuc....................................................................................................................................................   2   0   LT-3
....................................................................................aucugacaaaugcacuuc....................................................................................................................................................   2   0   JCK-3
....................................................................................aucugacaaaugcacuucaa..................................................................................................................................................   1   0   LT-3
....................................................................................aucugacaaaugcacuucaaagggaggGa.........................................................................................................................................   1   1   JCK-2
.....................................................................................ucugacaaaugcacuucaaagggaggGa.........................................................................................................................................   2   1   JCK-1
.....................................................................................ucugacaaaugcacuucaaagggaggGa.........................................................................................................................................   1   1   JCK-2
.....................................................................................ucugacaaaugcacuucaaagggaggGaag.......................................................................................................................................   1   1   JT-3
......................................................................................cugacaaaugcacuucaaagggaggGaagc......................................................................................................................................   1   1   JCK-1
......................................................................................cugacaaaugcacuucaaagggaggGaagc......................................................................................................................................   2   1   LT-2
......................................................................................cugacaaaugcacuucaaagggaggGaagc......................................................................................................................................   1   1   JCK-3
......................................................................................cugacaaaugcacuucaaagggaggGaagc......................................................................................................................................   2   1   LCK-3
......................................................................................cugacaaaugcacuucaaagggaggGaagc......................................................................................................................................   3   1   JCK-2
.......................................................................................ugacaaaugcacuucaaagggaggGaag.......................................................................................................................................   2   1   JT-3
.......................................................................................ugacaaaugcacuucaaagggaggGaagc......................................................................................................................................   1   1   JCK-3
.......................................................................................ugacaaaugcacuucaaagggaggGaagc......................................................................................................................................   1   1   LT-2
.......................................................................................ugacaaaugcacuucaaagggaggGaagc......................................................................................................................................   5   1   LCK-3
.......................................................................................ugacaaaugcacuucaaagggaggGaagc......................................................................................................................................   1   1   JCK-2
.......................................................................................ugacaaaugcacuucaaagggaggGaagc......................................................................................................................................   4   1   LT-1
.......................................................................................ugacaaaugcacuucaaagggaggGaagcu.....................................................................................................................................   4   1   JCK-3
.......................................................................................ugacaaaugcacuucaaagggaggGaagcu.....................................................................................................................................   7   1   LT-2
.......................................................................................ugacaaaugcacuucaaagggaggGaagcu.....................................................................................................................................   7   1   LT-1
.......................................................................................ugacaaaugcacuucaaagggaggGaagcu.....................................................................................................................................   9   1   JCK-1
.......................................................................................ugacaaaugcacuucaaagggaggGaagcu.....................................................................................................................................   2   1   JT-3
.......................................................................................ugacaaaugcacuucaaagggaggGaagcu.....................................................................................................................................   2   1   LCK-3
.......................................................................................ugacaaaugcacuucaaagggaggGaagcu.....................................................................................................................................   2   1   LT-3
.......................................................................................ugacaaaugcacuucaaagggaggGaagcu.....................................................................................................................................   2   1   JT-1
.......................................................................................ugacaaaugcacuucaaagggaggGaagcu.....................................................................................................................................   3   1   JCK-2
........................................................................................gacaaaugcacuucaaagggaggGa.........................................................................................................................................   5   1   LCK-3
........................................................................................gacaaaugcacuucaaagggaggGaag.......................................................................................................................................   1   1   LT-2
........................................................................................gacaaaugcacuucaaagggaggGaagc......................................................................................................................................   13  1   LCK-3
........................................................................................gacaaaugcacuucaaagggaggGaagc......................................................................................................................................   4   1   LT-1
........................................................................................gacaaaugcacuucaaagggaggGaagc......................................................................................................................................   10  1   JCK-2
........................................................................................gacaaaugcacuucaaagggaggGaagc......................................................................................................................................   6   1   LT-2
........................................................................................gacaaaugcacuucaaagggaggGaagc......................................................................................................................................   2   1   LT-3
........................................................................................gacaaaugcacuucaaagggaggGaagc......................................................................................................................................   6   1   JCK-3
........................................................................................gacaaaugcacuucaaagggaggGaagc......................................................................................................................................   8   1   JCK-1
........................................................................................gacaaaugcacuucaaagggaggGaagcu.....................................................................................................................................   7   1   JCK-3
........................................................................................gacaaaugcacuucaaagggaggGaagcu.....................................................................................................................................   11  1   JCK-2
........................................................................................gacaaaugcacuucaaagggaggGaagcu.....................................................................................................................................   2   1   JT-1
........................................................................................gacaaaugcacuucaaagggaggGaagcu.....................................................................................................................................   12  1   LT-3
........................................................................................gacaaaugcacuucaaagggaggGaagcu.....................................................................................................................................   2   1   JT-3
........................................................................................gacaaaugcacuucaaagggaggGaagcu.....................................................................................................................................   5   1   JCK-1
```

```
                  Mature                                                                                                 Star
cauuccggugcucaaucaaaaaaauaggccaugaaugaggaaguagugggccuuucacccucuuucuagugacucgaggagcugaucugacaaaugcacuucaaagggaggaaagcuagguuucccauguuaguauggccgggcauaaaagauuuugaaguuagguaaaaauugggaaggaugaaguaggaucuaugaaauaucgaagaaaguuccagucaacuucaaauggaguuuucgauccagggaa
........................................................................................gacaaaugcacuucaaagggaggGaagcu.....................................................................................................................................   14   1   LCK-3
........................................................................................gacaaaugcacuucaaagggaggGaagcu.....................................................................................................................................   8    1   LT-2
........................................................................................gacaaaugcacuucaaagggaggGaagcu.....................................................................................................................................   15   1   LT-1
........................................................................................gacaaaugcacuucaaagggaggGaagcua....................................................................................................................................   1    1   JCK-2
.........................................................................................acaaaugcacuucaaagggaggGaagc.......................................................................................................................................   1    1   LT-1
.........................................................................................acaaaugcacuucaaagggaggGaagc.......................................................................................................................................   2    1   JCK-2
.........................................................................................acaaaugcacuucaaagggaggGaagc.......................................................................................................................................   2    1   JCK-1
.........................................................................................acaaaugcacuucaaagggaggGaagc.......................................................................................................................................   3    1   JT-3
.........................................................................................acaaaugcacuucaaagggaggGaagc.......................................................................................................................................   3    1   JT-2
.........................................................................................acaaaugcacuucaaagggaggGaagc.......................................................................................................................................   3    1   JT-1
.........................................................................................acaaaugcacuucaaagggaggGaagc.......................................................................................................................................   1    1   LT-3
.........................................................................................acaaaugcacuucaaagggaggGaagcu......................................................................................................................................   1    1   LT-2
.........................................................................................acaaaugcacuucaaagggaggGaagcu......................................................................................................................................   6    1   JCK-1
.........................................................................................acaaaugcacuucaaagggaggGaagcu......................................................................................................................................   3    1   JT-2
.........................................................................................acaaaugcacuucaaagggaggGaagcu......................................................................................................................................   3    1   LCK-3
.........................................................................................acaaaugcacuucaaagggaggGaagcu......................................................................................................................................   6    1   JCK-2
.........................................................................................acaaaugcacuucaaagggaggGaagcu......................................................................................................................................   5    1   LT-1
.........................................................................................acaaaugcacuucaaagggaggGaagcu......................................................................................................................................   1    1   LT-3
.........................................................................................acaaaugcacuucaaagggaggGaagcu......................................................................................................................................   1    1   JT-3
.........................................................................................acaaaugcacuucaaagggaggGaagcu......................................................................................................................................   7    1   JCK-3
..........................................................................................caaaugcacuucaaagggaggGaagc.......................................................................................................................................   9    1   JCK-3
..........................................................................................caaaugcacuucaaagggaggGaagc.......................................................................................................................................   12   1   LT-1
..........................................................................................caaaugcacuucaaagggaggGaagc.......................................................................................................................................   3    1   LT-3
..........................................................................................caaaugcacuucaaagggaggGaagc.......................................................................................................................................   7    1   JCK-1
..........................................................................................caaaugcacuucaaagggaggGaagc.......................................................................................................................................   2    1   JT-1
..........................................................................................caaaugcacuucaaagggaggGaagc.......................................................................................................................................   7    1   LCK-3
..........................................................................................caaaugcacuucaaagggaggGaagc.......................................................................................................................................   8    1   LT-2
..........................................................................................caaaugcacuucaaagggaggGaagc.......................................................................................................................................   6    1   JCK-2
..........................................................................................caaaugcacuucaaagggaggGaagcu......................................................................................................................................   15   1   LCK-1
..........................................................................................caaaugcacuucaaagggaggGaagcu......................................................................................................................................   2    1   LT-3
..........................................................................................caaaugcacuucaaagggaggGaagcu......................................................................................................................................   19   1   LCK-3
..........................................................................................caaaugcacuucaaagggaggGaagcu......................................................................................................................................   5    1   JT-2
..........................................................................................caaaugcacuucaaagggaggGaagcu......................................................................................................................................   10   1   JCK-3
..........................................................................................caaaugcacuucaaagggaggGaagcu......................................................................................................................................   9    1   JT-3
..........................................................................................caaaugcacuucaaagggaggGaagcu......................................................................................................................................   2    1   JCK-1
..........................................................................................caaaugcacuucaaagggaggGaagcu......................................................................................................................................   16   1   LCK-2
..........................................................................................caaaugcacuucaaagggaggGaagcu......................................................................................................................................   2    1   JT-1
..........................................................................................caaaugcacuucaaagggaggGaagcu......................................................................................................................................   23   1   LT-2
..........................................................................................caaaugcacuucaaagggaggGaagcu......................................................................................................................................   7    1   JCK-2
..........................................................................................caaaugcacuucaaagggaggGaagcu......................................................................................................................................   13   1   LT-1
...........................................................................................................................cccauguuGguauggccgggca.........................................................................................................   2    1   LT-1
.............................................................................................................................cauguuGguauggccgggcaua.......................................................................................................   1    1   LCK-3
...................................................................................................................................Gguauggccgggcauaaaagauuuug.............................................................................................   1    1   LT-3
..........................................................................................................................................ccgggcauaaaagauuuugaag..........................................................................................   2    0   LT-1
..........................................................................................................................................ccgggcauaaaagauuuugaaUu.........................................................................................   1    1   LCK-3
..........................................................................................................................................ccgggcauaaaagauuuugaaguua.......................................................................................   2    0   JCK-2
..........................................................................................................................................ccgggcauaaaagauuuugaaguua.......................................................................................   1    0   JCK-3
..........................................................................................................................................ccgggcauaaaagauuuugaaguuaggua...................................................................................   1    0   LT-1
............................................................................................................................................gggcauaaaagauuuugaaguuaggu....................................................................................   1    0   LT-3
............................................................................................................................................gggcauaaaagauuuugaaguuaggua...................................................................................   1    0   LT-1
.....................................................................................................................................................agauuuugaaguuaggua...................................................................................   1    0   LT-3
..............................................................................................................................................................................ggaaggaugaaguaggaucuauga....................................................   1    0   JCK-3
..............................................................................................................................................................................ggaaggaugaaguaggaucuaugaaauau...............................................   2    0   JCK-2
...............................................................................................................................................................................gaaggaugaaguaggauc.........................................................   1    0   LT-3
...............................................................................................................................................................................gaaggaugaaguaggaucu........................................................   1    0   LT-3
...............................................................................................................................................................................gaaggaugaaguaggaucuaug.....................................................   2    0   LT-2
.................................................................................................................................................................................aggaugaaguaggaucua.......................................................   1    0   LCK-3
.................................................................................................................................................................................aggaugaaguaggaucuaugaa...................................................   2    0   JCK-2
.................................................................................................................................................................................aggaugaaguaggaucuaugaa...................................................   1    0   JCK-1
....................................................................................................................................................................................augaaguaggaucuaugaaauauc..............................................   1    0   LT-1
.....................................................................................................................................................................................ugaaguaggaucuaugaaau.................................................   2    0   LT-2
.....................................................................................................................................................................................ugaaguaggaucuaugaaauaucg.............................................   4    0   LCK-3
.....................................................................................................................................................................................ugaaguaggaucuaugaaauaucga............................................   1    0   LT-1
.....................................................................................................................................................................................ugaaguaggaucuaugaaauaucgaagaa........................................   1    0   JCK-1
......................................................................................................................................................................................gaaguaggaucuaugaaauaucgaagaU........................................   1    1   JCK-1
.......................................................................................................................................................................................aaguaggaucuaugaaauauc..............................................   1    0   JCK-2
.......................................................................................................................................................................................aaguaggaucuaugaaauauc..............................................   1    0   JCK-1
........................................................................................................................................................................................aguaggaucuaugaaauaucg.............................................   1    0   LT-3
........................................................................................................................................................................................aguaggaucuaugaaauaucgaag..........................................   6    0   JCK-3
........................................................................................................................................................................................aguaggaucuaugaaauaucgaag..........................................   4    0   JCK-2
```

```
                    Mature                                                                                                                       Star
cauuccggugcucaaucaaaaaaauaggccaugaaugaggaaguagugggccuuucacccucuuucuagugacucgaggagcugaucugacaaaugcacuucaaagggaggaaagcuagguuucccauguuaguauggccgggcauaaaagauuuugaaguuagguaaaaauugggaaggaugaaguaggaucuaugaaauaucgaagaaaguuccagucaacuucaaauggaguuuucgauccagggaa
........................................................................................................................................................................................aguaggaucuaugaaauaucgaag..........................................	4	0	JCK-1
.........................................................................................................................................................................................guaggaucuaugaaauaucga............................................	2	0	LT-1
...........................................................................................................................................................................................aggaucuaugaaauaucgaa...........................................	1	0	LT-3
...........................................................................................................................................................................................aggaucuaugaaauaucgaaga.........................................	2	0	LT-1
..................................................................................................................................................................................................augaaauaucgaagaaaguGc...................................	1	1	JCK-3
..................................................................................................................................................................................................augaaauaucgaagaaaguGc...................................	1	1	JCK-1
.......................................................................................................................................................................................................auaucgaagaaaguGccagu...............................	1	1	JCK-1
.......................................................................................................................................................................................................auaucgaagaaaguGccagu...............................	2	1	JCK-3
```
